# Telomeres and Estrogens: The Unholy Nexus in Pathogenesis of Atherosclerosis

**DOI:** 10.14740/cr335e

**Published:** 2014-07-20

**Authors:** Aga Syed Sameer, Saniya Nissar, Ruqaya Aziz

**Affiliations:** a Department of Biochemistry, Sher-I-Kashmir Institute of Medical Sciences Associated Medical College, Bemina, Srinagar, Kashmir 190018, India; b Department of Biochemistry, Kashmir University, Hazratbal, Srinagar, Kashmir 190006, India

**Keywords:** Atherosclerosis, Lipids, Telomeres, Lesion, HDL, LDL, Estrogens, Hormones, Arteries, Heart

## Abstract

Atherosclerosis (AS) is a complex inflammatory process and is categorized as a multifactorial disease involving the interplay of genetic and environmental factors. There are many factors which play role in predisposition and development of AS. In this review we have tried to address the basic pathophysiology of AS lesions and the role played by two important factors - telomeres and estrogens in the development of this disease.

## Introduction

Atherosclerosis (AS) is a multifaceted inflammatory process that involves the interaction of both adaptive and innate immune system of human body [1]. AS is categorized as a multifactorial disease in which genetic as well as environmental factors play a major role. AS is the single largest cause of death and disability in the western world [2]. AS is derived from the Greek words “athere” meaning gruel (accumulation of lipid) and “sclerosis” meaning hardening. AS is identified by the cholesterol deposition, macrophages infiltration, smooth muscle cells (SMCs) proliferation, connective tissue accumulation and thrombus formation [3]. A number of generalized or systemic factors have been identified as potential risk factors for the predisposition and development of AS. The disease is known to preferentially affect particular regions of the circulatory system especially heart and arteries. AS usually follows a well-defined progression in the circulatory system, usually in early stages the growth of the AS lesion is abluminal, and the progress may vary from total cessation in some cases [4]. AS starts early in life; AS appears first in the aorta (during fetal life) in its earliest stage, in the second decade of life it appears in the coronary arteries and in the third decade it makes its manifestation visible in the cerebral arteries [5].

The development and progression of AS as a disease is due to the interactions of numerous complex processes taking place in the individual [2, 6, 7]. AS lesions are actually the manifestation of the critical interactions of the various gene products of a number of patho-physiological pathways with the modulation by ambient environmental factors in a time-dependent manner. A number of processes are of central importance in the pathogenesis of AS especially endothelial cell (EC) dysfunction triggered by the atherogenic stimuli (i.e., increased plasma cholesterol and LDL levels), hypertension, diabetes and smoking. The presentation of the end points of AS has an astounding similarity among sufferers; however, the factors that have a role in influencing the progression of AS within an individual do not have any similarity [2].

## Classification of Atherosclerotic Lesion (AL)

According to the Committee on Vascular Lesions of the Council on Arteriosclerosis [8], the ALs have been classified into six types. This classification is based on a number of factors like the histological and histochemical composition, the structure and ultrastructure of both the cell and matrix components of the lesions. These types are described below.

Type I: This lesion is identified by the accumulation of atherogenic lipoproteins (apoB-containing lipoproteins) in the intimal space and infiltration of mononuclear leukocytes into the intimal space [1, 9]. This lesion is present at birth [2].

Type II: This lesion contains layers of macrophages or foam cells with SMC infiltration from the media into the intima of the vessel wall. The lesion has a distinguishing structure known as a “fatty streak” which is formed by the aggregation of foam cells and leukocytes within the intimal space. This is the earliest stage of AS which is visible to the naked eye and is unique to this stage of disease [1, 2, 8].

Type III: This lesion is an intermediary stage between types II and IV, which is identifiable by scattered coarse granules of lipid, accumulated foam cells or particles that disrupt the SMC integrity and distinct intimal thickening [1, 2].

Type IV: These lesions are characterized by “atheromas” which have a distinct structure of large extracellular lipid core and the abluminally growing AL. The extracellular lipid accumulation is termed as the lipid core. Severe intimal disorganization occurs due to the lipid core which appears to develop from an increase and the consequent union of the small secluded pools of extracellular lipid that characterizes type III lesion. Thus initially, atheroma is an eccentric lesion [1, 8]. In between the lipid core and the endothelial surface, the tissue layer is mostly intima that preceded lesion development. Between the lipid core and the endothelial surface, the intima contains macrophages and SMCs with and without the inclusions of lipids [8].

Type V: These lesions are characterized by atheromas containing large extracellular lipid cores and the developing of a prominent fibrous connective tissue which forms caps on or around the lipid core. This type of morphology may be referred to as fibroatheroma (Va lesion). A type V lesion can also be type Vb, in which the lipid core and other parts of the lesion are calcified and/or type Vc, in which a lipid core is absent and lipid in general is minimal. Type Va lesions are usually multilayered, having several lipid cores separated by thick layers of fibrous connective tissue [1, 8].

Type VI: These are complicated lesions with type IV/V morphology with ruptured atherosclerotic plaque. These are characterized by subsequent fissure formation or hematomas in the arterial lumen. These are usually formed when thrombogenic lipid core comes into contact with the blood resulting in platelet aggregation thus causing thrombosis. Type VI may be subdivided by the various special and common features. Thus, disruption of the surface may be labeled type VIa; hematoma or hemorrhage, type VIb; and thrombosis, type VIc. Type VIabc indicates the presence of all three features [1, 8].

## Telomeres and AS

In mammals, the ends of chromosomes contain a specialized region which protects the sub-telomeric region of the chromosome from wearing down and also prevents any abnormal associations between chromosomal ends. The telomeric DNA is composed of non-coding double stranded repeats of G-rich tandem DNA sequences (TTAGGG in vertebrates) [10]. These tandem repeats are extended several thousand base pairs (10 - 15 kb in humans and 25 - 40 kb in mice) and end in a 150 - 200 nucleotide 3’ single-stranded overhang. Telomeric DNA is also associated with several specific DNA binding proteins, which include telomerase and the telomeric repeat binding factors 1 and 2 (TRF1, TRF2) which associate with the TTAGGG repeat directly and interact with other factors forming large protein complexes that regulate telomere length and structure [11, 12]. Mammalian telomerase consists of an RNA component (telomerase RNA component (TERC)) that serves as a template for the synthesis of new telomeric TTAGGG repeats by the telomerase reverse transcriptase component (TERT) [10-12].

Functional telomeres are required for preserving genome integrity and stability by playing role in preventing the recognition of chromosomal ends as double-stranded DNA breaks [13, 14]. The protection of telomere depends on several factors, including the precise composition of telomere-associated proteins, the level of telomerase activity and telomere length itself [10]. Cells which contain sufficiently long telomeres do not require avid telomerase activity, but cells with critically short telomeres with low or lack of telomerase activity tend to have chromosomal fusions, replicative senescence and apoptosis [10, 11, 14]. The expression and activity of telomerase as well as the length of telomere which it synthesizes are both regulated in a tissue-specific and developmental manner in several species, including humans. During the embryonic development these parameters are greater and become low or undetectable after birth [15, 16]. In humans for instance, telomeric DNA length progressively shortens at a rate of 29 - 60 base pairs per year (bp/year) in many tissues like liver, renal cortex and spleen, but length of telomere remains stable in cerebral cortex [17]. In adult cell populations with high proliferative potential, human telomerase activity (hTERT) remains detectable, e.g. activated lymphocytes and certain types of stem cells [18-20]. Moreover, the occurrence of regulation of telomerase activity in tissue-specific manner, both during embryonic development and in adulthood is supported by an important body of evidences [21-23]. Accelerated rate of telomere attrition is characteristic feature of many premature aging syndromes like Werner syndrome, dyskeratosis congenital and ataxia telangectasia [14]. In several adult normal and tumor tissues, alternatively spliced human TERT (hTERT) transcripts have been detected and their expression as well has been shown to be regulated in a tissue-specific manner during development [24]. It has also led to the expression of hTERT isoforms which are known to lack functional reverse transcriptase domains, including dominant negative inhibitors of telomerase activity (e.g. hTERTα) [24-27].

Telomeres undergo wear and tear with each cycle of cellular replication until a critical telomere length is reached at which the cells experience replicative senescence. Thus, telomere length not only serves as an index of the replicative history of the cell but also is a key determinant of replicative senescence [28]. A number of observations are in support of this hypothesis which has been recently defined as the “telomere hypothesis of cellular aging” [29]. 1) Cancer cells, germline cells and immortalized cell lines usually express robust activity of telomerase, a reverse transcriptase. Thus, these types of cell continue to proliferate without evidence of replicative senescence and telomere erosion. 2) The ectopic expression of the catalytic subunit of telomerase in somatic cells expressing little or no telomerase activity results in the phenotypic transformation of these cells. 3) The inhibition of telomerase prevents the continuous growth of cancerous cells. 4) There is compelling evidence that reactive oxygen species (ROS) and sex are the two inter-related factors that accelerate the rate of telomere attrition in different cell types including vascular endothelial cells (VECs). 5) The telomere-mediated senescence of VECs may result in endothelial dysfunction particularly ALs.

Telomeres in essentiality record the replicative history and replicative potential of human somatic cells *in vitro* as well as *in vivo*. In human beings the telomere is relatively short, inversely correlated with age, highly heritable and longer in women than men. With each replication of somatic cells the telomere length becomes shorter and in cultured somatic cells from human beings. The “telomere hypothesis of cellular aging” states that telomeres serve as a “mitotic clock” of the cells [30]. Telomere attrition in cultured human somatic cells essentially follows the teleonomic behavior in that it ultimately leads to a well-defined outcome, i.e. a critically shortened telomere length signals replicative senescence. It is noteworthy that recent evidence indicates that replicative senescence may not be strictly related to telomere length but to the status of the telomeric DNA in relation to its telomeric protein complex, given that shortened telomeres appear to lose the protective shield of their binding proteins [29].

## Estrogens and AS

Estrogens represent the group of steroid compounds, named for their function in the estrous cycle, as primary female sex hormones. Estrogens exist in three forms: E2, estrone (E1) and estriol (E3). Among the estrogens E2 is the most essential compound as it is known to stimulate EC migration, proliferation and survival in ECs [31]. In the cardiovascular system, estrogen is activated by binding to estrogen receptors (ERs) in the ECs. There are basically two classical ER subtypes which are ERα and ERβ and a non-classical ER GPR30. ERs function in regulating the transcriptional processes in cells by binding to the ER in the nucleus which mediates dimerization and binding to specific response elements (EREs) to promote its target gene [32]. Estrogen mediates protein reactions within the cell by binding to the ER through protein-protein interactions with ERE in the nucleus. This stimulates the activator protein 1 (AP1) or SP1 sites in the promoter region of estrogen-responsive genes to activate coregulator proteins to the promoter, regulate mRNA levels and other protein production [31]. ER mediates the action of estrogens and the regulation of its gene expression by acting as ligand transcription factors. Estrogen is mediated selectively via ERα and ERβ in uterine artery and uterine artery endothelium. Physiological studies showed that ERα is more potent in stimulation of estrogen than ERβ ligands in VECs. ERα up-regulation in human dilated cardiomyopathy brings about increase in mRNA which was higher in women than men. The transcription factors of ER can interact with cytoplasmic proteins and activate signaling pathways [33, 34].

E2 stimulates ERs and HIF to the VEGF gene promoter in the endothelium. It has been observed that when the VEGF mRNA levels decline, ERα persists in its activity. HIF1 mediates phosphatidyl inositol 3-kinase (PI3K) pathway in estrogen induction of VEGF expression in the endometrium [31, 35]. Estrogen also mediates the signaling of tissue factory pathway inhibitor-I (TFPI) of blood coagulation. TFPI has been found to be involved in angiogenesis due to its stimulation with ER ligands. The regulation of TFPI involves post-transcriptional effects mediated by the one version of ERα which is amino-terminally truncated with molecular weight of 45 kDa. Its involvement in angiogenesis is due to its stimulation with ER ligands [36, 37]. TFPI may affect angiogenesis through peptides within its carboxyl terminus which may directly block VEGF2 activation, thereby hindering the migration of ECs [38].

Estrogen mediates its cardioprotective effect via ER-mediated non-genomic signaling pathways [37]. Membrane ER binding results in rapid, non-genomic actions and is mediated by several pathways, some important of which are receptor tyrosine kinases and protein kinases including PI3K, Akt, mitogen-activated protein kinase, Src protein kinase A and C and by increasing the concentration of intracellular calcium [39]. With regard to cardiovascular events, direct membrane signaling causes vasodilatation through nitric oxide release and opening of the calcium-activated potassium channels through an NO and cyclic GMP pathway [39]. A number of studies have observed that acute addition of 17β-estradiol to either ovary-intact females or ovariectomized females reduces the avidity of ischemic reperfusion. Some studies suggest location of ERs at the plasma membrane, where they could elicit rapid protective effects via the activation of non-genomic signaling pathways [40].

Estrogen critically binds at different to initiate acute signaling pathway. It alters different levels of proteins and signaling pathways, leading to post-translational modifications that alter protein activities [41]. A direct protein-protein interaction between ligand-activated ERα and the regulatory subunit p85 of PI3K in ECs through a non-genomic mechanism by which E2 rapidly stimulates eNOS via the activation of PI3K/Akt would lead to downstream activation of NOS/NO/SNO signaling. This clearly suggested that ERα activation of PI3K might play a role in cardio-protection [42]. NOS can be signaled via S-nitrosylation (SNO) and SNO levels are mediated by activation of estrogen. Some research work also suggested that ERβ agonist could increase the SNO of a series of proteins in ovariectomized females [43].

Estrogens usually exert a protective effect against ROS-induced DNA damage, because of its anti-oxidant properties [44]. Estrogens are also known to induce TERT transcription, and hence enhancement of the telomerase activity, in MCF-7 cells via an estrogen response element within the TERT promoter [45]. Estrogens also stimulate the phosphatidylinositol 3 kinase/Akt pathway via post-transcriptional modifications of a number of mediators including Akt protein kinase [28, 46, 47]. Estrogens also stimulate nitric oxide production in VECs, which in turn has been found to stimulate the telomerase activity in these cells [28].

Numerous evidences have been provided both *in vitro* and *in vivo* for direct effects of estrogens on telomerase activity. First, in the endometrium telomerase activity varies with the stage of the menstrual cycle [48], being negligible during menstruation but increases during the follicular phase, reaching maximum levels immediately before ovulation, coinciding with a peak in estrogen levels and proliferative activity, and then falls during the luteal phase. Postmenopausal endometrium and endometrium from women treated with antiestrogens exhibit decreased telomerase activity [12]. 

## References

[1] Singh RB, Mengi SA, Xu YJ, Arneja AS, Dhalla NS (2002). Pathogenesis of atherosclerosis: A multifactorial process. Exp Clin Cardiol.

[2] Hegele RA (1997). The genetic basis of atherosclerosis. Int J Clin Lab Res.

[3] Turunen MP, Hiltunen MO, Yla-Herttuala S (1999). Gene therapy for angiogenesis, restenosis and related diseases. Exp Gerontol.

[4] Libby P, Schoenbeck U, Mach F, Selwyn AP, Ganz P (1998). Current concepts in cardiovascular pathology: the role of LDL cholesterol in plaque rupture and stabilization. Am J Med.

[5] Napoli C, D'Armiento FP, Corso G, Ambrosio G, Palumbo G, Zuliani P, Malorni A (1997). Occurrence of the same peroxidative compounds in low density lipoprotein and in atherosclerotic lesions from a homozygous familial hypercholesterolemic patient: a case report. Int J Cardiol.

[6] Fuster V, Badimon L, Badimon JJ, Chesebro JH (1992). The pathogenesis of coronary artery disease and the acute coronary syndromes (1). N Engl J Med.

[7] Ross R (1993). The pathogenesis of atherosclerosis: a perspective for the 1990s. Nature.

[8] Stary HC, Glagov S, Newman WP, Schaffer SA (1990). Changes in the cells of atherosclerotic lesions as advanced lesions evolve in coronary arteries of children and young adults. Pathobiology of the human atherosclerotic plaque.

[9] Nordestgaard BG, Wootton R, Lewis B (1995). Selective retention of VLDL, IDL, and LDL in the arterial intima of genetically hyperlipidemic rabbits in vivo. Molecular size as a determinant of fractional loss from the intima-inner media. Arterioscler Thromb Vasc Biol.

[10] Blasco MA (2005). Telomeres and human disease: ageing, cancer and beyond. Nat Rev Genet.

[11] Blackburn EH (2001). Switching and signaling at the telomere. Cell.

[12] Fuster JJ, Andres V (2006). Telomere biology and cardiovascular disease. Circ Res.

[13] Blasco MA (2003). Mammalian telomeres and telomerase: why they matter for cancer and aging. Eur J Cell Biol.

[14] Edo MD, Andres V (2005). Aging, telomeres, and atherosclerosis. Cardiovasc Res.

[15] Cherif H, Tarry JL, Ozanne SE, Hales CN (2003). Ageing and telomeres: a study into organ- and gender-specific telomere shortening. Nucleic Acids Res.

[16] Prowse KR, Greider CW (1995). Developmental and tissue-specific regulation of mouse telomerase and telomere length. Proc Natl Acad Sci U S A.

[17] Takubo K, Izumiyama-Shimomura N, Honma N, Sawabe M, Arai T, Kato M, Oshimura M (2002). Telomere lengths are characteristic in each human individual. Exp Gerontol.

[18] Hiyama K, Hirai Y, Kyoizumi S, Akiyama M, Hiyama E, Piatyszek MA, Shay JW (1995). Activation of telomerase in human lymphocytes and hematopoietic progenitor cells. J Immunol.

[19] Chiu CP, Dragowska W, Kim NW, Vaziri H, Yui J, Thomas TE, Harley CB (1996). Differential expression of telomerase activity in hematopoietic progenitors from adult human bone marrow. Stem Cells.

[20] Liu K, Schoonmaker MM, Levine BL, June CH, Hodes RJ, Weng NP (1999). Constitutive and regulated expression of telomerase reverse transcriptase (hTERT) in human lymphocytes. Proc Natl Acad Sci U S A.

[21] Blasco MA, Funk W, Villeponteau B, Greider CW (1995). Functional characterization and developmental regulation of mouse telomerase RNA. Science.

[22] Ulaner GA, Giudice LC (1997). Developmental regulation of telomerase activity in human fetal tissues during gestation. Mol Hum Reprod.

[23] Martin-Rivera L, Herrera E, Albar JP, Blasco MA (1998). Expression of mouse telomerase catalytic subunit in embryos and adult tissues. Proc Natl Acad Sci U S A.

[24] Ulaner GA, Hu JF, Vu TH, Giudice LC, Hoffman AR (1998). Telomerase activity in human development is regulated by human telomerase reverse transcriptase (hTERT) transcription and by alternate splicing of hTERT transcripts. Cancer Res.

[25] Ulaner GA, Hu JF, Vu TH, Giudice LC, Hoffman AR (2001). Tissue-specific alternate splicing of human telomerase reverse transcriptase (hTERT) influences telomere lengths during human development. Int J Cancer.

[26] Yi X, White DM, Aisner DL, Baur JA, Wright WE, Shay JW (2000). An alternate splicing variant of the human telomerase catalytic subunit inhibits telomerase activity. Neoplasia.

[27] Saeboe-Larssen S, Fossberg E, Gaudernack G (2006). Characterization of novel alternative splicing sites in human telomerase reverse transcriptase (hTERT): analysis of expression and mutual correlation in mRNA isoforms from normal and tumour tissues. BMC Mol Biol.

[28] Aviv A (2002). Chronology versus biology: telomeres, essential hypertension, and vascular aging. Hypertension.

[29] Aviv A (2002). Telomeres, sex, reactive oxygen species, and human cardiovascular aging. J Mol Med (Berl).

[30] Harley CB, Vaziri H, Counter CM, Allsopp RC (1992). The telomere hypothesis of cellular aging. Exp Gerontol.

[31] Barnabas O, Wang H, Gao XM (2013). Role of estrogen in angiogenesis in cardiovascular diseases. J Geriatr Cardiol.

[32] Bjornstrom L, Sjoberg M (2005). Mechanisms of estrogen receptor signaling: convergence of genomic and nongenomic actions on target genes. Mol Endocrinol.

[33] Jin X, Chen YC, Liu WQ, Wang HY, Wang B, Zeng Z (2008). [Estradiol promote myocardial angiogenesis in a rat model of acute myocardial infarction through estrogen receptors]. Sichuan Da Xue Xue Bao Yi Xue Ban.

[34] Mahmoodzadeh S, Eder S, Nordmeyer J, Ehler E, Huber O, Martus P, Weiske J (2006). Estrogen receptor alpha up-regulation and redistribution in human heart failure. FASEB J.

[35] Albrecht ED, Babischkin JS, Lidor Y, Anderson LD, Udoff LC, Pepe GJ (2003). Effect of estrogen on angiogenesis in co-cultures of human endometrial cells and microvascular endothelial cells. Hum Reprod.

[36] Dahm AE, Iversen N, Birkenes B, Ree AH, Sandset PM (2006). Estrogens, selective estrogen receptor modulators, and a selective estrogen receptor down-regulator inhibit endothelial production of tissue factor pathway inhibitor 1. BMC Cardiovasc Disord.

[37] Holroyd EW, Delacroix S, Larsen K, Harbuzariu A, Psaltis PJ, Wang L, Pan S (2012). Tissue factor pathway inhibitor blocks angiogenesis via its carboxyl terminus. Arterioscler Thromb Vasc Biol.

[38] O'Lone R, Knorr K, Jaffe IZ, Schaffer ME, Martini PG, Karas RH, Bienkowska J (2007). Estrogen receptors alpha and beta mediate distinct pathways of vascular gene expression, including genes involved in mitochondrial electron transport and generation of reactive oxygen species. Mol Endocrinol.

[39] Manavathi B, Nair SS, Wang RA, Kumar R, Vadlamudi RK (2005). Proline-, glutamic acid-, and leucine-rich protein-1 is essential in growth factor regulation of signal transducers and activators of transcription 3 activation. Cancer Res.

[40] Deschamps AM, Murphy E, Sun J (2010). Estrogen receptor activation and cardioprotection in ischemia reperfusion injury. Trends Cardiovasc Med.

[41] Murphy E (2011). Estrogen signaling and cardiovascular disease. Circ Res.

[42] da Silva MB, Farges RC, Frode TS (2004). Involvement of steroids in anti-inflammatory effects of PK11195 in a murine model of pleurisy. Mediators Inflamm.

[43] Lin J, Steenbergen C, Murphy E, Sun J (2009). Estrogen receptor-beta activation results in S-nitrosylation of proteins involved in cardioprotection. Circulation.

[44] Mooradian AD (1993). Antioxidant properties of steroids. J Steroid Biochem Mol Biol.

[45] Kyo S, Takakura M, Kanaya T, Zhuo W, Fujimoto K, Nishio Y, Orimo A (1999). Estrogen activates telomerase. Cancer Res.

[46] Kang SS, Kwon T, Kwon DY, Do SI (1999). Akt protein kinase enhances human telomerase activity through phosphorylation of telomerase reverse transcriptase subunit. J Biol Chem.

[47] Breitschopf K, Zeiher AM, Dimmeler S (2001). Pro-atherogenic factors induce telomerase inactivation in endothelial cells through an Akt-dependent mechanism. FEBS Lett.

[48] Tanaka M, Kyo S, Takakura M, Kanaya T, Sagawa T, Yamashita K, Okada Y (1998). Expression of telomerase activity in human endometrium is localized to epithelial glandular cells and regulated in a menstrual phase-dependent manner correlated with cell proliferation. Am J Pathol.

